# Inhibition of the HCV Core Protein on the Immune Response to HBV Surface Antigen and on HBV Gene Expression and Replication In Vivo

**DOI:** 10.1371/journal.pone.0045146

**Published:** 2012-09-14

**Authors:** Wenbo Zhu, Chunchen Wu, Wanyu Deng, Rongjun Pei, Yun Wang, Liang Cao, Bo Qin, Mengji Lu, Xinwen Chen

**Affiliations:** 1 State Key Lab of Virology, Wuhan Institute of Virology, Chinese Academy of Sciences, Wuhan, China; 2 Institute of Virology, University Hospital of Essen, Essen, Germany; Pohang University of Science and Technology, Republic of Korea

## Abstract

The hepatitis C virus (HCV) core protein is a multifunctional protein that can interfere with the induction of an immune response. It has been reported that the HCV core protein inhibits HBV replication in vitro. In this study, we test the effect of the HCV core gene on the priming of the immune response to hepatitis B surface antigen (HBsAg) and on the replication of HBV in vivo. Our results showed that the full-length HCV core gene inhibits the induction of an immune response to the heterogeneous antigen, HBsAg, at the site of inoculation when HCV core (pC191) and HBsAg (pHBsAg) expression plasmids are co-administered as DNA vaccines into BALB/c mice. The observed interference effect of the HCV core occurs in the priming stage and is limited to the DNA form of the HBsAg antigen, but not to the protein form. The HCV core reduces the protective effect of the HBsAg when the HBsAg and the HCV core are co-administered as vaccines in an HBV hydrodynamic mouse model because the HCV core induces immune tolerance to the heterogeneous HBsAg DNA antigen. These results suggest that HCV core may play an important role in viral persistence by the attenuation of host immune responses to different antigens. We further tested whether the HCV core interfered with the priming of the immune response in hepatocytes via the hydrodynamic co-injection of an HBV replication-competent plasmid and an HCV core plasmid. The HCV core inhibited HBV replication and antigen expression in both BALB/c (H-2d) and C57BL/6 (H-2b) mice, the mouse models of acute and chronic hepatitis B virus infections. Thus, the HCV core inhibits the induction of a specific immune response to an HBsAg DNA vaccine. However, HCV C also interferes with HBV gene expression and replication in vivo, as observed in patients with coinfection.

## Introduction

Hepatitis C virus (HCV) infection causes chronic hepatic inflammation in patients and severe liver diseases, such as liver cirrhosis and hepatocellular carcinoma [1], [2]. Approximately 170 million people worldwide are infected with HCV. This has been largely attributed to the inability of the host immune system to clear the initial HCV infection [3], [4]. No vaccine against HCV is available despite intensive research efforts.

The HCV core protein (C) is an RNA-binding, dimeric, alpha-helical protein that participates in the formation of the HCV viral nucleocapsid. It is the most conserved protein among the six major HCV genotypes [5], [6]. Current data have shown that the HCV C may be one of the main proteins responsible for the capability of HCV to escape immune detection [7]. The HCV core protein interacts with a variety of cellular proteins to influence host cell functions and can affect innate and adaptive immune pathways [8], [9], [10]. The HCV core inhibits type I IFN production by interacting with intracellular viral recognition receptors, such as Toll-like receptors and helicases [11], and regulates the function of NF-κB, which is involved in both innate and adaptive immunity [12]. Moreover, the HCV core protein impairs the in vitro priming, activation and proliferation of T cells [13], [14], [15], [16], [17], [18], [19].

Various vaccines based on the HCV C have failed to induce a strong immune response, even though the vaccines contain several well-characterised epitopes that can be recognised by B cells and cytotoxic T lymphocytes (CTLs) [20], [21], [22], [23], [24], [25], [26], [27]. Earlier studies using a full-length HCV core DNA sequence have revealed a limited immunogenicity of the naked DNA [28], as increasing doses or repeated administration of the full-length HCV core DNA were unable to enhance the specific immune responses [29]. Interestingly, a vector expressing a truncated version of the HCV C improved the induction of specific T- and B-cell responses to the HCV C [28], [29], [30]. A previous study from our lab demonstrated that the full-length HCV C inhibited the priming of the immune response by the truncated version of the HCV C using a DNA vaccination strategy [29].The observed interference may be explained by the dysfunction of dendritic cells that are interacting with the full-length HCV C locally at the inoculated sites [13], [31], [32]. It is not yet known whether the HCV C can interfere with specific immune responses to the heterogeneous antigens such as hepatitis B virus (HBV) proteins during a co-infection with HCV and HBV.

It has been reported that a number of patients may be co-infected with HCV and HBV [33], [34]. Early clinical studies have shown that HCV interferes with HBV replication, leading to the suppression of HBV replication or even to HBV clearance [35], [36], [37], [38]. Other in vitro studies have suggested that the HCV C may specifically participate in the suppression of HBV replication [39], [40].

In this study, we investigated whether the HCV C interfered with the induction of specific immune responses to heterogeneous antigens. We chose the HBV surface antigen (HBsAg) as the model antigen, and our results revealed that the full-length HCV C was able to inhibit the induction of a specific immune response to an HBsAg DNA vaccine challenge. The interference of the HCV C with HBsAg was specific for the HBsAg DNA antigen, but not for the HBsAg recombinant protein antigen. To determine whether the HCV C exerted an inhibitory effect on HBV gene expression and replication, we used an HBV mouse model based on hydrodynamic injection (HI) [41], [42]. Our results showed that the co-administration of the HCV core with HBV replication-competent plasmid pAAV-HBV1.3 inhibited HBV replication and gene expression in vivo; this finding is consistent with clinical findings. These data indicated that the inhibitory effect of the HCV C on HBV was the dominant factor influencing the immune outcome. Our strategy using the HBV mouse model based on HI will allow for further analysis of the interactions between HBV and HCV proteins.

## Results

### 1. Co-administration of pC191 and pHBsAg Prevented the Effective Induction of an Immune Response to HBsAg

**Figure 1:**
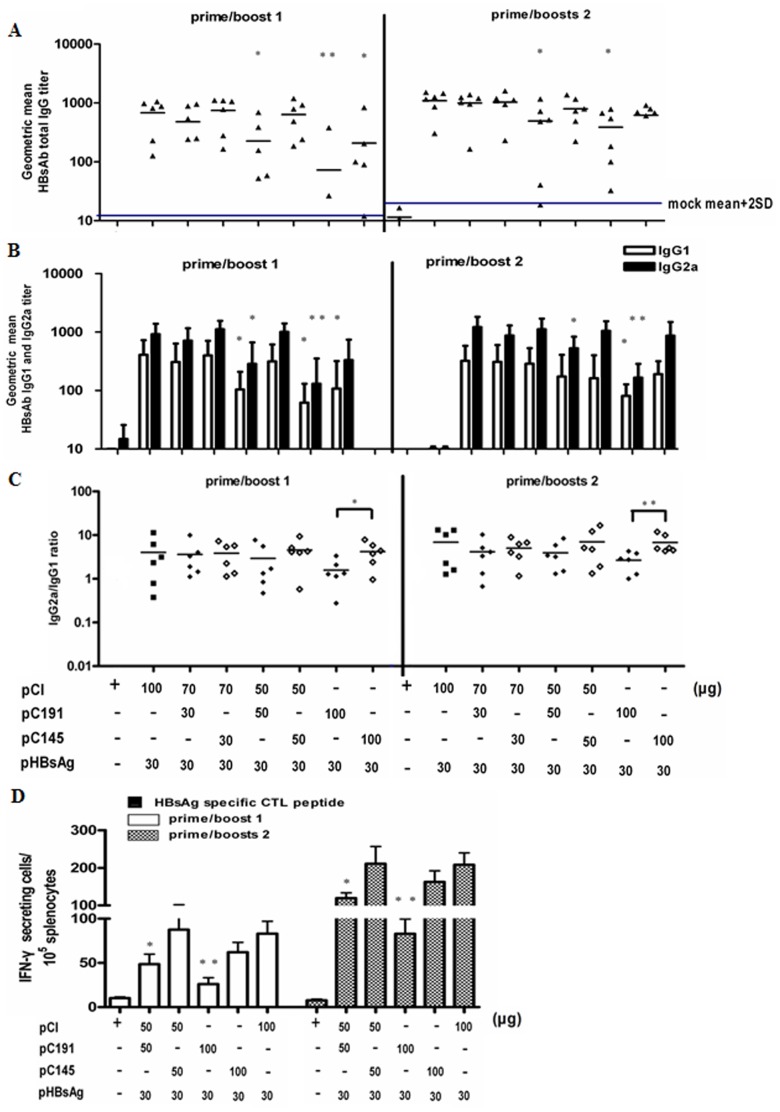
Co-administration of pC191 and pHBsAg prevented effective priming of immune responses to HBsAg. BALB/c mice (n = 6 for each group) were immunised three times with different concentrations and different combinations of the pHBsAg, pCI-neo (pCI), pC191, or pC145 constructs by in vivo electroporation. Sera from 6 mice per group were collected on the 10^th^ day after the first or second immunisation boost and were serially diluted and titred by ELISA. (A) Total HBsAb IgG. (B) HBsAb IgG subclasses IgG1 and IgG2a. (C) Ratios of HBsAb IgG2a/IgG1. (D) Splenocytes were collected at day 10 after the third immunization and subjected to ELISPOT assay. Splenocytes were re-stimulated with an HBsAg peptide (H-2L^d^ CTL epitope aa 29–38). HBsAg specific IFN-producing cells were determined by identified by spot formation. The numbers represent the means of spot-forming cells per 2×10^5^ splenocytes. The error bars represent the standard deviation. Statistically significant differences between the groups are displayed as *(p<0.05) or **(p<0.01).

In our previous study, we demonstrated that co-administration of DNA vaccines expressing the full-length and truncated HCV core gene resulted in the inhibition of an HCV core-specific immune response [29]. Therefore, we investigated whether the co-administration of expression plasmids encoding the full-length HCV C could also inhibit specific immune responses to a heterogeneous antigen. DNA vaccines expressing the full-length HCV C (pC191) or a truncated version of the HCV C (pC145) and the HBsAg (pHBsAg) were administered to BALB/c mice (H-2^d^), and the immune responses to HBsAg were assessed. The HBsAb levels in mice after two or three immunisations were determined by Enzyme-linked immunosorbent assay (ELISA) (Fig. 1A–C). Our results showed that the co-administration of pHBsAg (30 µg) with pC191 (both 50 µg and 100 µg) led to a decreased HBsAb response when compared with the control group co-administered with pCI-neo (100 µg) and pHBsAg (30 µg) (Fig. 1A), whereas co-administration of 30 µg of pC191 had no significant effect on HBsAb responses. Co-administration of pHBsAg and pC145 at the doses 30 and 50 µg did not reduced the HBsAb response in mice but at 100 µg after two immunisations (p<0.05) (Fig. 1A).

The subtypes of the HBsAb IgG antibodies were further analysed. IgG2a is the predominant subtype induced by intramuscular DNA immunisation. Co-administration of pHBsAg (30 µg) and pC191 (both 50 µg and 100 µg) resulted in a reduction in both HBsAb IgG1 and IgG2a responses as compared with the control group co-administered with pCI-neo (100 µg) and pHBsAg (30 µg) (Fig. 1B). The co-administration of pC145 at the doses of 30 and 50 µg had no effect on HBsAb IgG1 and -2 responses. Only the co-administration of a high dose of pC145 (100 µg) resulted in lower HBsAb IgG1 levels after two immunisations (p<0.05). The Th1/Th2 ratio was next assessed among the different immunisation groups (Fig. 1C). Compared with groups that received pHBsAg co-administered with either pCI-neo or pC145, the group that received pHBsAg (30 µg) and pC191 (100 µg) developed an HBsAb response with a lower IgG2a/IgG1 ratio, suggesting that the full-length HCV C may preferentially inhibit the induction of the Th1 response.

The cell-mediated immune response to HBsAg was assessed using the enzyme-linked immunosorbent spot (ELISPOT) assay. All groups exhibited T cell responses to the HBsAg peptide (aa29-38: IPQSLDSWWTSL, an H-2L^d^-restricted CD8+ T cell epitope) following two immunisations (Fig. 1D). Co-administration of pC191 and pHBsAg led to a significant reduction in the numbers of IFN-γ-producing cells when compared with the control group co-administered with pCI-neo and pHBsAg. The results after three immunisations showed the same trend.

These data indicated that pC191 inhibited the induction of HBsAg-specific immune responses, whereas pC145, encoding a truncated HCV C, showed a partial inhibition of HBsAg immune responses only at a high dose.

**Figure 2:**
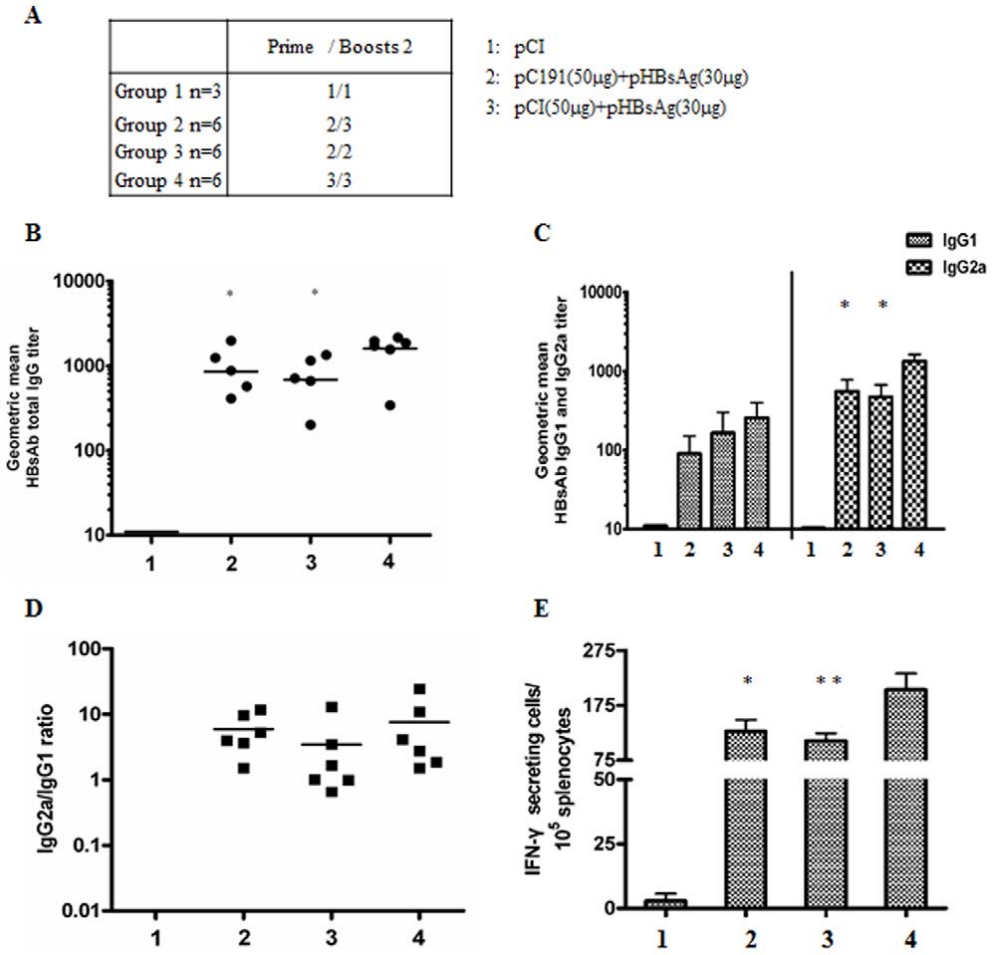
Co-application of pC191 inhibited the effective boosting of HBsAg-specific immune response. BALB/c mice (n = 6 for each group) were immunized three times by in vivo electroporation with different doses of the plasmids pCI, pHBsAg, pC191, or combinations as indicated in (A). Sera of immunized mice were collected on 10th days after the third immunization, serially diluted, and tested by ELISA assay. (B) Total HBsAb IgG responses. (C) HBsAb IgG subclasses IgG1 and IgG2a. (D) Ratios of HBsAb IgG2a/IgG1. (E) Splenocytes were collected from mice at day 10 after the third immunization and subjected to ELISPOT assay. Splenocytes were re-stimulated with a HBsAg peptide (H-2L^d^ CTL epitope aa 29–38). HBsAg specific IFN-producing cells were determined by identified by spot formation. The numbers represent the means of spot-forming cells per 2×10^5^ splenocytes. The error bars represent the standard deviation. Statistically significant differences between the groups are displayed as *(p<0.05) or **(p<0.01).

To further test the interference of the full-length HCV core, BALB/c mice were immunised twice with pC191 and pHBsAg and then boosted with pHBsAg by in vivo electroporation (Fig. 2A). The results showed that the mice in the group 2 that received the final immunisation with pHBsAg displayed similar anti-HBs levels as the group 3 that was co-immunised three times with pC191 and pHBsAg. The anti-HBs levels in both groups (group 2 and group 3) were significantly lower than those of the group immunised three times with pHBsAg (p<0.05) (Fig. 2B). Analysis of the IgG subclasses showed that there was no significant difference in the HBsAg IgG1 levels in all of the treatment groups. Co-administration of pC191 and pHBsAg resulted in a lower level of the HBsAb IgG2a subtype compared with group 4 (p<0.05). An immunisation boost with pHBsAg after co-administration of pC191 and pHBsAg did not increase the HBsAb IgG2a titre, which was significantly lower than that of the group immunised with pHBsAg only (p<0.05) (Fig. 2C). However, there was no significant difference in the IgG2a/IgG1 ratio among all of the groups (Fig. 2D). The ELISPOT assays showed that the boost with pHBsAg after co-administration of pC191 and pHBsAg resulted in lower levels of IFN-γ-producing cells than the group that was immunised only with pHBsAg (p<0.01) but a similar number of IFN-γ-producing cells as the group that was co-administered pC191 and pHBsAg (Fig. 2E). These results indicated that boosting with the heterogeneous DNA antigen, HBsAg, did not induce a stronger immune response upon re-challenge with the same antigen.

In conclusion, co-administration of the expression vector of HCV C interfered only with HCV C-specific B and T-cell responses, but also generally with the initiation of immune responses to other proteins like HBsAg.

### 2. The Full-length HCV Core did not Prevent Priming of Immune Responses to HBsAg when pC191 and pHBsAg were Administered at Different Sites

**Figure 3:**
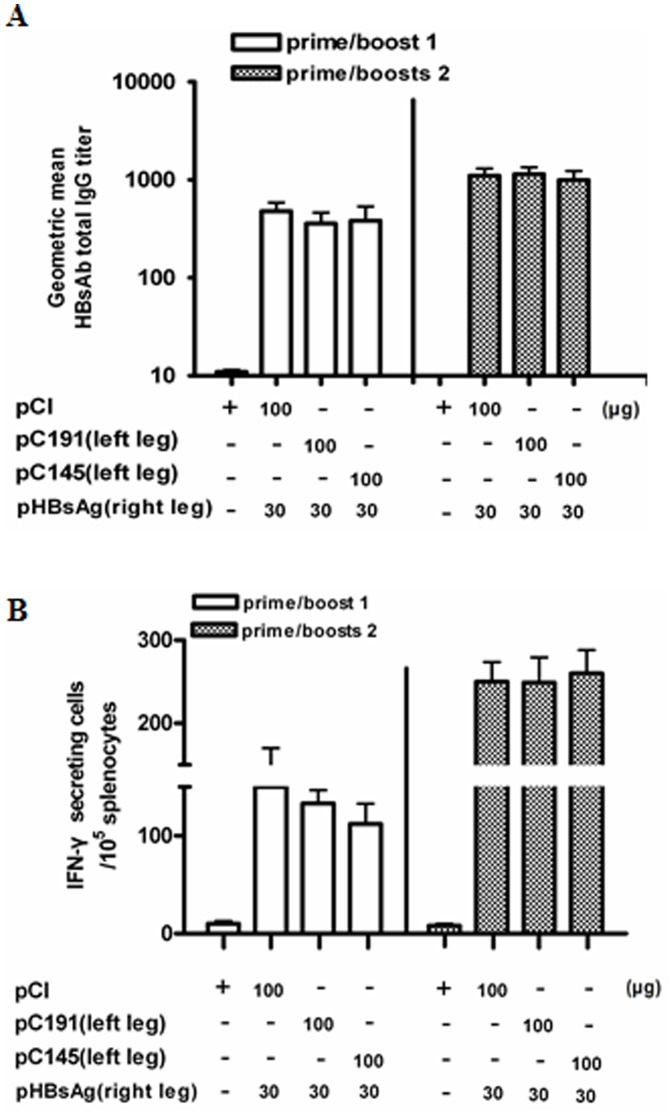
Co-application of pC191 at the different sites cannot prevent the priming of immune responses to HBsAg. BALB/c mice (n = 6 for each group) were immunized three times by in vivo electroporation with different plasmids at different leg. Sera of immunized mice were collected on the 10^th^ day after second and third immunizations, serially diluted, and testd by ELISA assay. (A) Total HBsAb IgG responses. Splenocytes were collected from mice at day 10 after the third immunization and subjected to ELISPOT assay. (B) Splenocytes were re-stimulated with an HBsAg peptide (H-2L^d^ CTL epitope aa 29–38).HBsAg specific IFN-producing cells were determined by identified by spot formation. The numbers represent the means of spot-forming cells per 2×10^5^ splenocytes. The error bars represent the standard deviation. Statistically significant differences between the groups are displayed as *(p<0.05) or **(p<0.01).

To test whether pC191 (expressing the full-length HCV C) prevented the priming of specific immune responses by DNA vaccines administered at different sites, BALB/c mice were simultaneously injected in the left leg with pC191, p145, or pCI and in the right leg with pHBsAg by electroporation. The HBsAb titres and the frequencies of T cells responsive to the HBsAg peptide (aa 29–38) were determined by ELISA and ELISPOT, respectively. The HBsAb levels and the frequency of spot-forming cells were similar for all groups, whether they were immunised two or three times with pHBsAg (Fig. 3A and B); this finding indicates that the co-administration of pC191 and pHBsAg prevented the effective priming of immune responses only when they were administered at the same site. The full-length HCV C thus exerts its immunosuppressive action locally, but not systemically. Thus, HCV C expressed in the liver during HCV infection may play a role for HCV persistence by the attenuation of host immune responses to HCV C itself and other HCV proteins.

### 3. pC191 did not Prevent the Effective Priming of Immune Responses Induced by rHBsAg Antigen

**Figure 4:**
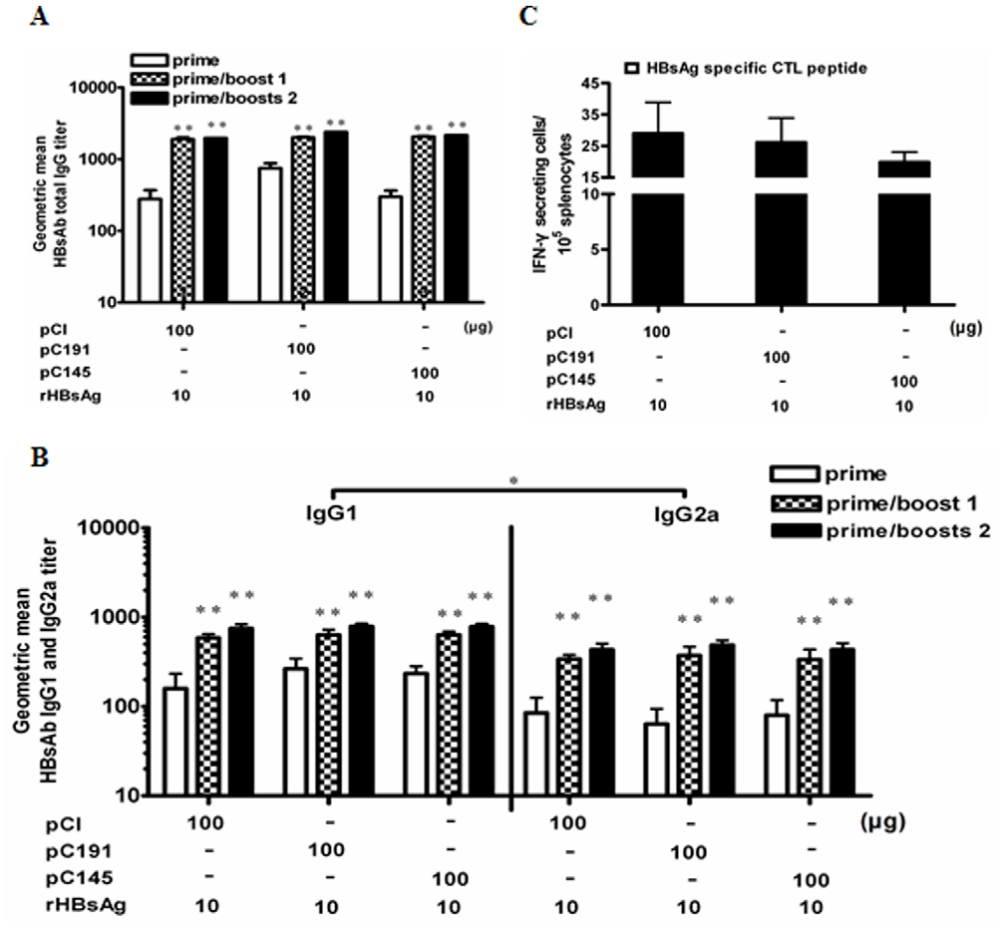
Co-administration of pC191 and rHBsAg cannot prevent priming of immune responses to recombinant HBsAg. BALB/c mice (n = 6 for each group) were immunised three times by in vivo electroporation with different combinations of plasmid and/or recombinant HBsAg (rHBsAg). Sera of immunized mice were collected on the 10th day after the first or second immunisation boost and were serially diluted and titred by ELISA. (A) Total HBsAb IgG. (B) HBsAb IgG subclasses IgG1 and IgG2a. (C) Splenocytes were collected from mice at day 10 after the third immunization and subjected to ELISPOT assay. Splenocytes were re-stimulated with an HBsAg peptide (H-2L^d^ CTL epitope aa 29–38). HBsAg specific IFN-producing cells were determined by identified by spot formation. The numbers represent the means of spot-forming cells per 2×10^5^ splenocytes. The error bars represent the standard deviation. Statistically significant differences between the boost and the boosts relative to the prime in each group are displayed as *(p<0.05) or **(p<0.01).

Next, we asked whether the full-length HCV core gene interfered with the priming of specific immune responses to the recombinant protein antigen. BALB/c mice were immunised three times with the HCV C expression plasmids, pC191 or pC145, and recombinant HBsAg (rHBsAg) without adjuvant by electroporation (Fig. 4).All three groups of mice developed the same level of HBsAb and T cell responses. The second and third immunisations significantly increased the total IgG, IgG1 and IgG2a HBsAb titres (p<0.01), and the HBsAb IgG1 response was significantly higher than the HBsAb IgG2a response in all of the treatment groups (p<0.05). However, there was no significant difference in the HBsAb among the three groups. Thus, pC191 and pC145 did not influence the priming of the immune response to rHBsAg, suggesting that the full-length HCV C is able to interfere with the priming of the immune response by plasmid DNA, but not by protein antigen.

### 4. Clearance of HBV after HI of pAAV/HBV1.3 in Mice Immunised with pHBsAg with and without pC191

**Figure 5:**
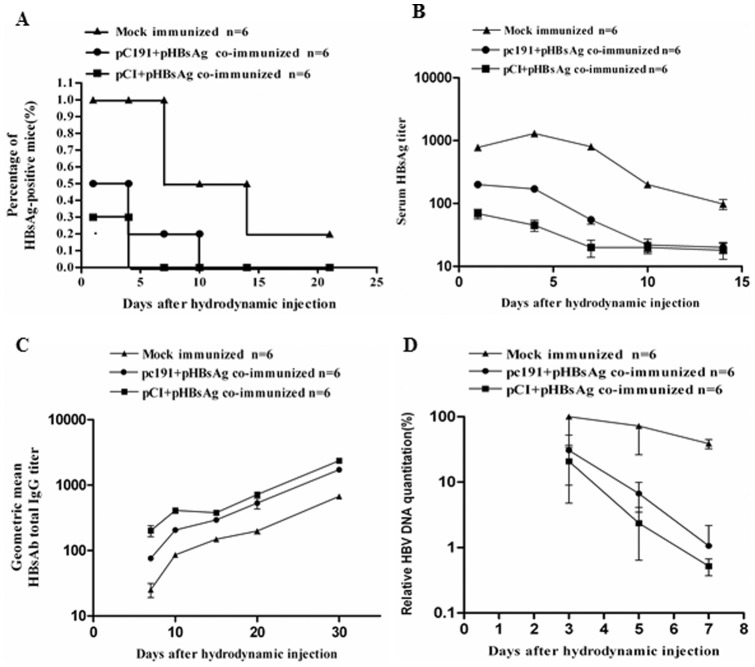
The co-administration of pC191 with pHBsAg delayed HBsAg and HBV DNA clearance in immunized mice after HI. BALB/c (H-2^d^) mice (n = 6 for each group) were immunized three times by in vivo electroporation with different plasmid combinations and then challenged with pAAV/HBV1.3 by tail vein HI on day 5 after the third immunization. Sera were collected at the indicated time points for HBsAg, HBsAb, and HBV DNA detection. (A) The positive rate of serum HBsAg in mice of each group. (B) The kinetic of the HBsAg titres. (C) The kinetics of the total HBsAb IgG titres. (D) The kinetics of HBV DNA detection in mice. The results shown represent the average of all of the mice.

Given that the co-administration of pC191 with pHBsAg inhibited the priming of HBsAg-specific immune responses, we sought to determine whether the clearance of HBV might be impaired in pre-immunised hosts. The HBV mouse model based on hydrodynamic injection (HI) is a useful system to study HBV clearance after the vaccination against HBV proteins [43].Thus, BALB/c (H-2^d^) mice were immunised and challenged with pAAV/HBV1.3 by HI (Fig. 5). All mice in the pCI-neo mock immunisation group were HBsAg-positive after 7 days post HI (dphi), and thereafter, they gradually cleared HBsAg (Fig. 5A). One mouse remained HBsAg-positive at 21 dphi. Only 2 of 6 mice co-immunised with pCI-neo and pHBsAg were HBsAg-positive on 1 dphi, and all mice cleared HBsAg by 4 dphi (Fig. 5A). In the group co-immunised with pC191 and pHBsAg, 3 of 6 mice showed HBsAg serological conversion by 1 dphi, and all mice cleared the HBsAg before 10 dphi. Consistently, the pCI-neo mock immunisation group developed high HBsAg levels within 1 week after HI, and the levels gradually decreased thereafter. The titres of HBsAg in the mock group were higher than those in the other treatment groups at all-time points during the follow-up period, and the lowest HBsAg titres were detected in the mice co-immunised with pCI-neo and pHBsAg (p<0.01) (Fig. 5B). The kinetics of the HBsAb response in mice following HI showed the same trends (Fig. 5C). The serum HBsAb titres in mice co-immunised with pCI-neo and pHBsAg were higher than the levels in mice co-immunised with pC191 and pHBsAg (p<0.05) prior to 15 dphi. Both groups showed significantly higher serum HBsAb titres than the mock immunisation group (p<0.05). The quantification of HBV DNA in mouse sera showed similar results (Fig. 5D).

These results indicated that the pHBsAg-immunised mice cleared the HBsAg faster than the mock-immunised mice. However, the co-administration of pC191 decreased the protective immune responses against HBV induced by pHBsAg, indicating that the HCV C may impair the priming of effective immune responses and delay antigen clearance. These findings demonstrated that the attenuation of host immune responses is not only measurable by in vitro assays but had influences on the course of viral clearance in vivo, suggesting again the important role of HCV C expression for HCV persistence.

### 5. The Full-length HCV C Inhibited HBV Replication and Reduced the HBsAg Level in vivo after HI

**Figure 6:**
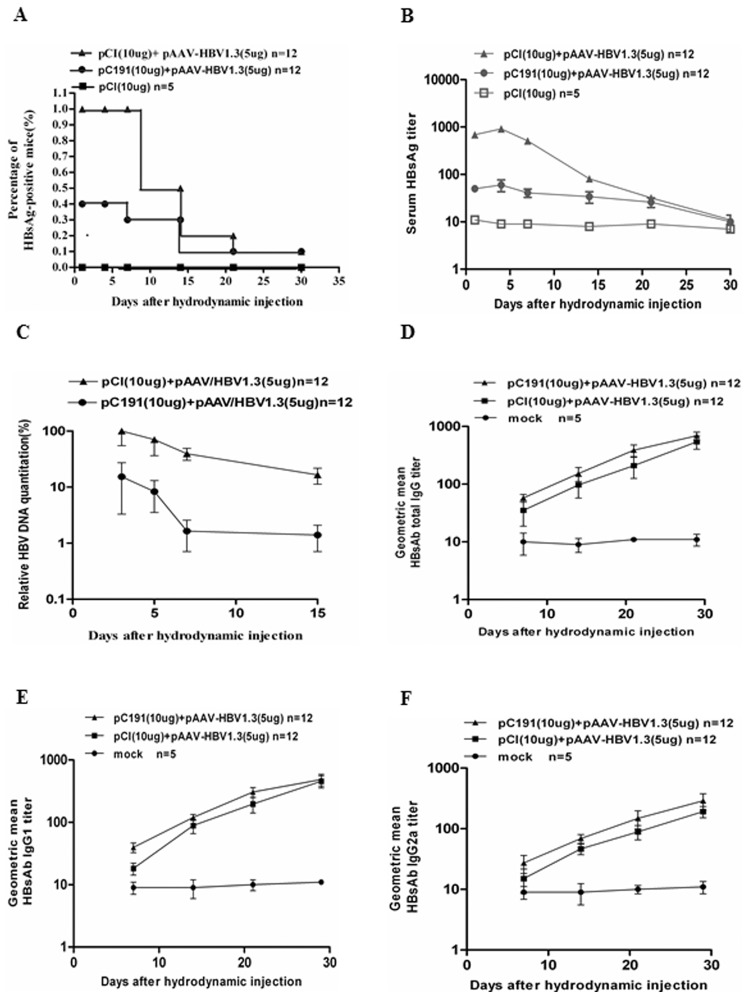
Co-administration of pC191 and pAAV/HBV1.3 by HI reduced HBV replication and gene expression in vivo. BALB/c (H-2^d^) mice (n = 12 for each group) were subjected to HI with different plasmids combinations. Sera were collected and subjected to HBsAg and HBV DNA detection at the indicated time points. The kinetics of HBsAb total IgG and IgG subclasse responses after HI in BALB/c (H-2^d^) mice were tested by ELISA assay. (A) Positive rate of serum HBsAg in the mice from each group. (B) The kinetics of the HBsAg titer after HI. (C) The kinetics of HBV DNA detection in the mice. The kinetics of the total HBsAb IgG (D), IgG1 (E) and IgG2a (F) titres after HI. The results shown represent the average of all of the mice. The error bars represent the standard deviations. Statistically significant differences between the groups are displayed as *(p<0.05) and **(p<0.01).

Previous studies have shown that the HCV C interferes with HBV replication and gene expression in vitro [39], [40](Fig. S1). Thus, we asked how the expression of the HCV C influenced HBV replication and gene expression in vivo. As the HCV C also interfered with the priming of HBsAg-specific immune responses, we could not exclude the possibility that the immune clearance of HBV in the liver was impaired. BALB/c (H-2^d^) mice were subjected to HI with pAAV/HBV1.3 in the presence of pCI-neo or pC191. The serum HBsAg titres in the mice were determined at the indicated time points by ELISA. All mice in the group that received pAAV/HBV1.3 and pCI-neo were HBsAg-positive until 7 dphi. The number of HBsAg-positive mice decreased thereafter, consistent with previous reports [42](Fig. 6A). In contrast, 7 of the 12 mice that received pAAV/HBV1.3 and pC191 remained negative for HBsAg. Only 5 mice were positive for HBsAg at 4 dphi and showed a gradual decrease in the serum HBsAg levels.

Furthermore, the serum HBsAg concentrations in mice received pAAV/HBV1.3 and pCI-neo were significantly higher than that in mice administered pAAV/HBV1.3 and pC191(p<0.01) before 15 dphi. Thereafter, both groups had decreased but similar HBsAg levels by 20 dphi and became HBsAg negative by 30 dphi (Fig. 6B). It suggests that the expression of the HCV C may decrease HBsAg expression in mice.

The quantitative analysis of the serum HBV DNA concentration revealed that the group administered pAAV/HBV1.3 and pC191 showed lower serum HBV DNA levels and a more rapid clearance of serum HBV DNA when compared with the control group administered pAAV/HBV1.3 and pCI (p<0.05) (Fig. 6C). By 42 dphi, all mice cleared serum HBV DNA (data not shown).

The titres of the HBsAb IgG subclasses in mice were determined by ELISA at the indicated time points after HI. The titres of HBsAb IgG and IgG1 in mice administered pAAV/HBV1.3 and pC191 were higher than the corresponding levels in the control group administered pAAV/HBV1.3 and pCI from 7 to 21 dphi (p<0.05), but they reached the same level at 30 dphi (Fig. 6D and E). Similarly, the titres of HBsAb IgG2a in mice administered pAAV/HBV1.3 and pC191 were significantly higher than the IgG2a levels in the control mice until 30 dphi (p<0.01) (Fig. 6F). The results shown represent the average of all of the mice.

These data indicated that the interference of the HCV C with the priming of HBsAg-specific immune responses was only observed if both the HCV C and the HBsAg expression plasmids were administered at the same anatomic site. In an acute HBV infection mouse model, HCV core could not impair the immune clearance of HBV in the liver. Inversely HCV C could inhibit HBV replication and reduce the HBsAg level in vivo, which may be partly due to its effect on promoting the anti-HBs antibody response in BALB/c (H-2^d^) mice after HI.

### 6. The Full-length HCV Core Reduced HBsAg Levels and Promoted the Anti-HBs Antibody Response in a Mouse Model of Persistent HBV Replication

**Figure 7:**
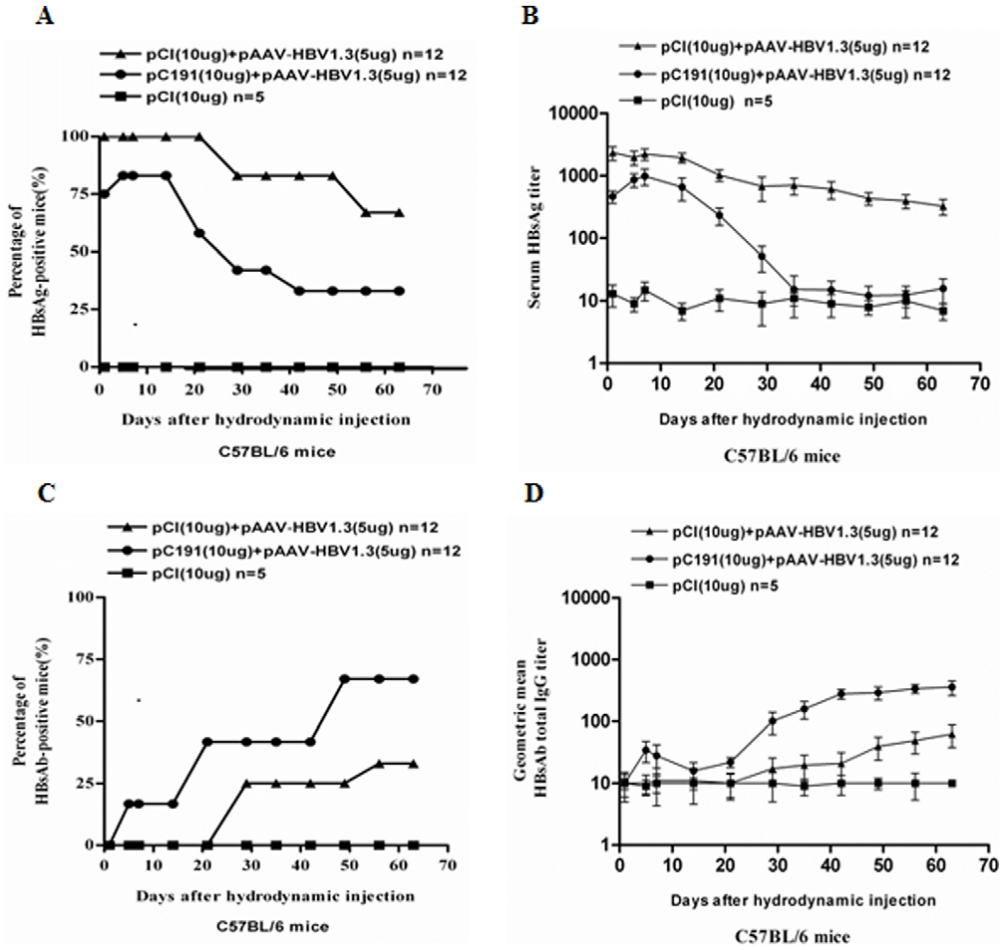
The HCV core reduced HBsAg levels and promoted the HBsAb antibody response in a mouse model of persistent HBV replication. C57BL/6 (H-2^b^) mice (n = 12 for each group) were challenged with different plasmids combinations by tail vein HI. Sera were collected at indicated time points, serially diluted, and tested by ELISA assay.(A) Positive rate of serum HBsAg in the mice from each group. (B) The kinetics of the HBsAg titres after HI. (C) Positive rate of serum HBsAb in the mice from each group. (D) The kinetics of the total HBsAb IgG titres after HI. The error bars represent the standard deviation. Statistically significant differences between the groups are displayed as *(p<0.05) and **(p<0.01).

Huang et al. established a mouse model of persistent HBV replication after HI in the H-2^b^ haplotype mouse strain, C57BL/6 [41], [42]. We thus used this model to determine how the expression of the full-length HCV core might affect HBV persistence. We performed HI in C57BL/6 (H-2^b^) mice with pAAV/HBV1.3 in the presence of pC191 or pCI. All mice of the group received pAAV/HBV1.3 and pCI were HBsAg positive before the 21st dphi and 67% of mice were still HBsAg-positive at 60 dphi. In contrast, mice received pAAV/HBV1.3 and pC191 produced only transient antigenemia and 70% of mice lost HBsAg already at 42 dphi (Fig. 7A). The serum HBsAg titers in mice co-injected with pAAV/HBV1.3 and pCI were significantly higher than that in mice co-injected with pAAV/HBV1.3 and pC191 at all the indicated time points (p<0.01) (Fig. 7B). Furthermore, the serum HBsAg concentrations in mice injected with pAAV/HBV1.3 and pC191 decreased to the same level as the mock group by 35 dphi (Fig. 7B). None of mice receiving pAAV/HBV1.3 and pCI developed HBsAb at 21 dphi and only 30% of mice were HBsAb-positive after the 56th dphi. 60% of mice receiving pAAV/HBV1.3 and pC191 developed HBsAb within 49 dphi (Fig. 7C). The titer of HBsAb IgG in the sera from mice received pC191 and pAAV/HBV1.3 was significantly higher than that with pCI and pAAV/HBV1.3 from 30 to 60 dphi (p<0.01) (Fig. 7D).

These data consistently indicated that the HCV C reduces the HBsAg expression and facilitated HBV clearance in vivo and promote HBsAb response. These findings were consistent with that in patients with HCV-HBV co-infection. Thus, The HBV mouse model is a valuable model for the depth studies in the future, particularly to identify the molecular and immunological mechanisms underlying our findings.

## Discussion

In the present study, we examined the administration of a vector expressing the HCV C on the development of specific immune responses to the HBV surface antigen and on HBV gene expression and replication in an HBV mouse model using HI. The strategy employed in our study will help provide novel insights into the pathogenesis of HBV-HCV co-infection and will allow us to study the actions of HCV proteins on HBV gene expression and replication as well as the pathogenesis of HBV infection.

In a previous study, we showed that the plasmid DNA expressing the full-length HCV C inhibited the priming of T and B cell immune responses to a truncated HCV C [29]. In the current study, we demonstrated that the full-length HCV C also interfered with the induction of specific immune responses to HBsAg, a heterologous protein. Notably, vaccinations with recombinant HBsAg protein were not affected by vaccination with the HCV core gene. These results indicated that the HCV C preferentially interfered with the initiation of immune responses at an early stage. Zimmermann et al. have reported that the HCV C impairs the in vitro priming of T cell responses by dendritic cells and hepatocytes [19]. The negative effect of the HCV C on the function of antigen presenting cells was observed whether the HCV C was expressed intracellularly with an expression vector or whether it was administered exogenously to hepatocytes in the form of a recombinant protein [19]. Consistent with these results, our data demonstrated that the interference of the HCV C with the priming of HBsAg-specific immune responses was only observed if both the HCV C and the HBsAg expression plasmids were administered at the same anatomic site. An additional control experiment showed that the co-administration of the expression vectors did not reduce the expression levels of HBsAg in vitro (Fig. S2).

The ability of the HCV C to interfere with the priming of immune responses has implications in the design of HCV vaccines or certain vaccine combinations. High expression levels of the HCV C would not induce stronger immune responses but would instead inhibit the priming of immune responses, as shown in the previous studies [29], [44]. The interference of the HCV C with the priming of immune responses to other antigens may reduce the efficacy of combined genetic vaccines. A rational vaccine design might use the truncated HCV C, as it demonstrated improved immunogenicity; the truncated HCV C induced specific T- and B-cell responses in a dose-dependent manner [28], [29], [30]. Our present study demonstrated that the truncated HCV C at a high does retains a residual activity to interfere with the priming of specific immune responses to other antigens.

Consistent with previous publications, the expression of the HCV C had an inhibitory effect on HBV gene expression and replication in vitro [39], [40]. By contrast, other studies have indicated that the HCV C may attenuate host virus-specific immune responses [19], [45], therefore generally impair viral clearance in vivo. In this study, we used an HI-based HBV mouse model [41], [42] and co-administered an expression vector coding for the HCV C with the HBV clone, pAAV/HBV1.3. Our results showed that the HCV C could inhibit HBV gene expression and replication in mouse models of both acute and chronic HBV infection. The inhibition of HBV gene expression and replication may facilitate and accelerate the clearance of HBV in mice though, HCV C may weaken the immune response to HBV at the same time. As the sum of the different factors, the impairment of host immune responses to HBV by the expression of the HCV C was not pronounced in HBV mouse models.

The prevalence of HCV infection in patients with HBV infection has been examined in several studies [33], [34]. Some clinical studies have shown that HCV exerts an interference effect that suppresses or terminates the HBV carrier state [35], [36], [37], [38]. Other findings have suggested a reciprocal inhibition between these two viruses in patients who are co-infected with HBV and HCV [46], [47], [48].

Chen et al. have demonstrated that the HCV C can suppress HBV gene expression by directly interacting with the trans-activator HBX protein [39]. Moreover, the HCV C can suppress HBV replication by forming a complex with the HBV polymerase (pol). Our study demonstrated for the first time the suppressive effect of the HCV C on HBV gene expression and replication in vivo. However, there are three papers dealing with the HBV-HCV co-infection studies demonstrated that there is no direct interference in virus level [49], [50], [51]. These discrepancys maybe due to differences in the co-infection system. The paper of Bellecave et al did not show the influence of HCV on HBV, as their cell lines could not be infected by HCV properly [49]. Hiraga et al used a cell line with stably HBV replication [50]. It is more difficult to get any effect on HBV if a high replication level is pre-established. In our experiment, we used transient transfection. If we start HBV replication by transfection, it is more susceptible to any inhibitory influence. The work of Eyre et al explored Ad-mediated HBV replication that may be more robust than transfection [51].

## Materials and Methods

### Ethics Statements

This study was carried out in strict accordance with the recommendations in the Guide for the Care and Use of Laboratory Animals according to the regulation in the People's Republic of China. The protocol was approved by the official Committee on the Ethics of Animal Experiments of Wuhan Institute of Virology, Chinese Academy of Science ((License number: SYXK2005-0034). Female BALB/c mice (6–8 weeks old; H-2^d^) and female C57BL/6 mice (6–8 weeks old; H-2^b^) were raised under specific pathogen-free conditions in the Central Animal Laboratory of the institute. All procedures were performed under isoflurane anesthesia, and all efforts were made to minimize suffering.

### Constructs and Recombinant Proteins

The pC191 and pC145 plasmids were constructed using cDNA from an HCV genotype 1a isolate [29]. The regions from nucleotide (nt) 1 to 572 or from nt 1 to 434 encoding the HCV core protein were amplified by PCR using primers tagged with *Eco*RI and *Xba*I restriction sites. The PCR products were cleaved with *Eco*RI and *Xba*I restriction enzymes and inserted into the corresponding sites of the eukaryotic expression vector, pCI-neo, generating the plasmids, pC191 and pC145. The pHBsAg expression plasmid containing the HBV S gene (nt 160 to 841 of the HBV subtype *adw,* GenBank accession no. AF282918) was constructed as previously described [52].An HBV replication-competent plasmid pAAV-HBV1.3 was used for HI (GenBank accession no. X02763.1, subtype *adw*) [53].

Recombinant HBsAg (rHBsAg) from HBV subtype *adw* (400 µg/ml in PBS) was kindly provided by Bing Yan of the Wuhan Institute of Virology in China. The rHBsAg was expressed in yeast and had a purity >98%.

### Plasmid DNA and Protein Immunisation

Plasmid DNA was prepared using the Qiagen Plasmid Maxi Kit (Qiagen, Germany) according to the manufacturer’s instructions. Mice were randomly divided into control or experimental groups. In vivo electroporation was performed according to a protocol described previously [29]. Mice were immunised with the indicated amount of plasmid DNA dissolved in 50 µl of TE buffer. When rHBsAg and plasmid DNA were used simultaneously, 10 µg of rHBsAg and 10 µg of one of the plasmids were dissolved in 50 µl of TE buffer and administered intramuscularly by electroporation into the quadriceps of the mice. Two booster immunisations were administered at time intervals of 3 weeks after the initial immunisation. Mice were sacrificed 10 days after the final immunisation. Splenocytes from the vaccinated mice were prepared for analysis by enzyme-linked immunosorbent spot (ELISPOT) assay. Sera were collected and stored at −20°C.

### The Mouse Model of HI

Mice were challenged by HI of pAAV/HBV1.3 into the tail vein as described previously [42]. Each mouse received 5 µg of the pAAV/HBV1.3 plasmid and/or 10 µg of the pC191 plasmid in a volume of PBS equivalent to 8% of the body weight of the mouse. The entire dose was delivered within 5 to 8 sec.

### Detection of HBV Antigen and HBsAb by ELISA in Serum

At the indicated time points following immunisation, serum samples were collected from mice via retro-orbital bleeding. The titres of HBsAg and HBsAb in mouse sera were measured by ELISA. Microtitre plates pre-coated with HBsAg were purchased commercially (Kehua, Shanghai, China). Serial dilutions (50 µl at dilutions ranging from 1∶10 to 1∶2,560) of mouse sera were added to the wells and incubated for 1 h at 37°C. Mouse IgG, IgG1, or IgG2a antibodies that bound to HBsAg antigen were detected with the following secondary antibodies conjugated to horseradish peroxidase: goat anti-mouse IgG (1∶1,000; Novagen, USA), goat anti-mouse IgG1 (1∶1,000; SouthernBiotech, USA) or goat anti-mouse IgG2a (1∶1,000; SouthernBiotech, USA), respectively. ELISA plates were developed at room temperature and read on a spectrophotometer at 450 nm. HBsAg was also detected using serial dilutions (1∶10 to 1∶2,560) of mouse sera with the commercial HBsAg EIA kit (Kehua, Shanghai, China) according to the manufacturer’s instructions. The EIA values were read on a spectrophotometer at 450 nm. Standard ELISA assays were performed for the final quantification of the titres of HBsAg and anti-HBs antibodies in all of the test sera from each individual experiment. The titres were calculated based on the sample dilution that yielded an OD450 of 0.300 using the appropriate standards and controls. Certain samples were re-assayed to ensure consistency of the results and to render the experiments comparable to each other.

### Detection of Serum HBV DNA

Serum samples were collected at the indicated time points after HI of pAAV/HBV1.3. For the detection of HBV DNA, each serum sample was pre-treated with 30 units of DNase I (Roche, Mannheim, Germany) and incubated overnight at 37°C. Total DNA was extracted and subjected to real-time PCR to quantify the HBV DNA. The real-time PCR experiments were performed using the DNA Master SYBR Green kit (Roche, Mannheim, Germany) in the Roche Light cycler V.3 for 40 cycles each with the following cycling parameters: 95°C for 20 s, 57°C for 15 s, and 72°C for 10 s. The pAAV/HBV1.3 plasmid was diluted and used as a reference standard. The specificity of the PCR products was verified using a melting curve analysis and agarose gel electrophoresis. To determine the geometric mean of serum HBV DNA copies in the mice, serum from the mock group was designated as 100% at 3 dphi, and then, the relative HBV DNA (%) in each sample was calculated at 3,5, and 7 dphi in each group.

### Enzyme-Linked Immunospot (ELISPOT) Assay

The ELISPOT assay was performed using the mouse IFN-γ pre-coated ELISPOT Kit (Dakewe, Shenzhen, China) according to the manufacturer’s instructions. Briefly, 96-well flat-bottomed microtitre plates were pre-incubated with the coating antibody (an anti-IFN-γ monoclonal antibody) overnight at 4°C and then blocked for 2 h at 37°C. Mouse splenocytes at 2×10^5^ cells per well were added in triplicate and then incubated with 2 µg/ml of the HBsAg peptide (aa29-38: IPQSLDSWWTSL, an H-2L^d^-restricted CD8+ T cell epitope) (Shanghai Sangon, China) at 37°C in 5% CO_2_ for 24 h. ConA was used at a concentration of 5 µg/ml (Sigma, St. Louis, USA) as a positive control. After incubation with the HBsAg peptide, the cells were washed ten times with PBS containing 0.05% Tween-20 and then incubated with 100 µl of a biotinylated anti-IFN-γ antibody for 1 h. The plates were washed again with PBST and incubated with 50 µl of HRP-streptavidin solution at 37°C for 1 h. Spot-forming cells were counted and analysed on an ELISPOT plate reader (BioReader 4000, Biosys, Germany). The results were presented as spot-forming cells per 2×10^5^ cells.

### Statistical Analysis

Statistical analysis was conducted using GraphPad software (San Diego, USA). The Student’s t-test was used to compare the results between the different groups. P values <0.05 were considered to represent statistically significant differences. Data are presented as the means ± standard deviation.

## Supporting Information

Figure S1 **HBV replication and gene expression in Huh7 cells in the presence of HCV core protein.** Huh7 cells were co-transfected with plasmids of pSM2 and pC191 or pCI-neo. The HBsAg and HBeAg titers in the culture supernatants (A, B) and cell lysats (C, D) were determined. The amounts of encasidated HBV DNA in transfected cells were determined by real time PCR (E).
(PDF)

Figure S2 **HBsAg expression in hepatoma cells in the presence of HCV core protein.** HepG2 cells were co-transfected with plasmids pCI-neo, pHBsAg, or pC191 as indicated. HBsAg in the culture supernatants (A) and cell lysats (B) of transfected HepG2 cells were determined.
(PDF)

Figure S3 **Immunohistochemistry staining analysis of HBsAg and HCV in mice liver.** BALB/c (H-2d) mice were subjected to HI with different plasmids combinations. Liver were collected and subjected to immunohistochemistry staining with anti-HBsAg (Thermo) (A, C and D) and serum of HCV patient (B, D and F) at 72 h (magnification: 200 X ).
(PDF)
