# Supplementary figures and images for: Inhibition of the HCV Core Protein on the Immune Response to HBV Surface Antigen and on HBV Gene Expression and Replication In Vivo
Source: PLoS One. 2012 Sep 14;7(9):e45146. doi: 10.1371/journal.pone.0045146 (PMC3443233; doi:10.1371/journal.pone.0045146)

Figure S1

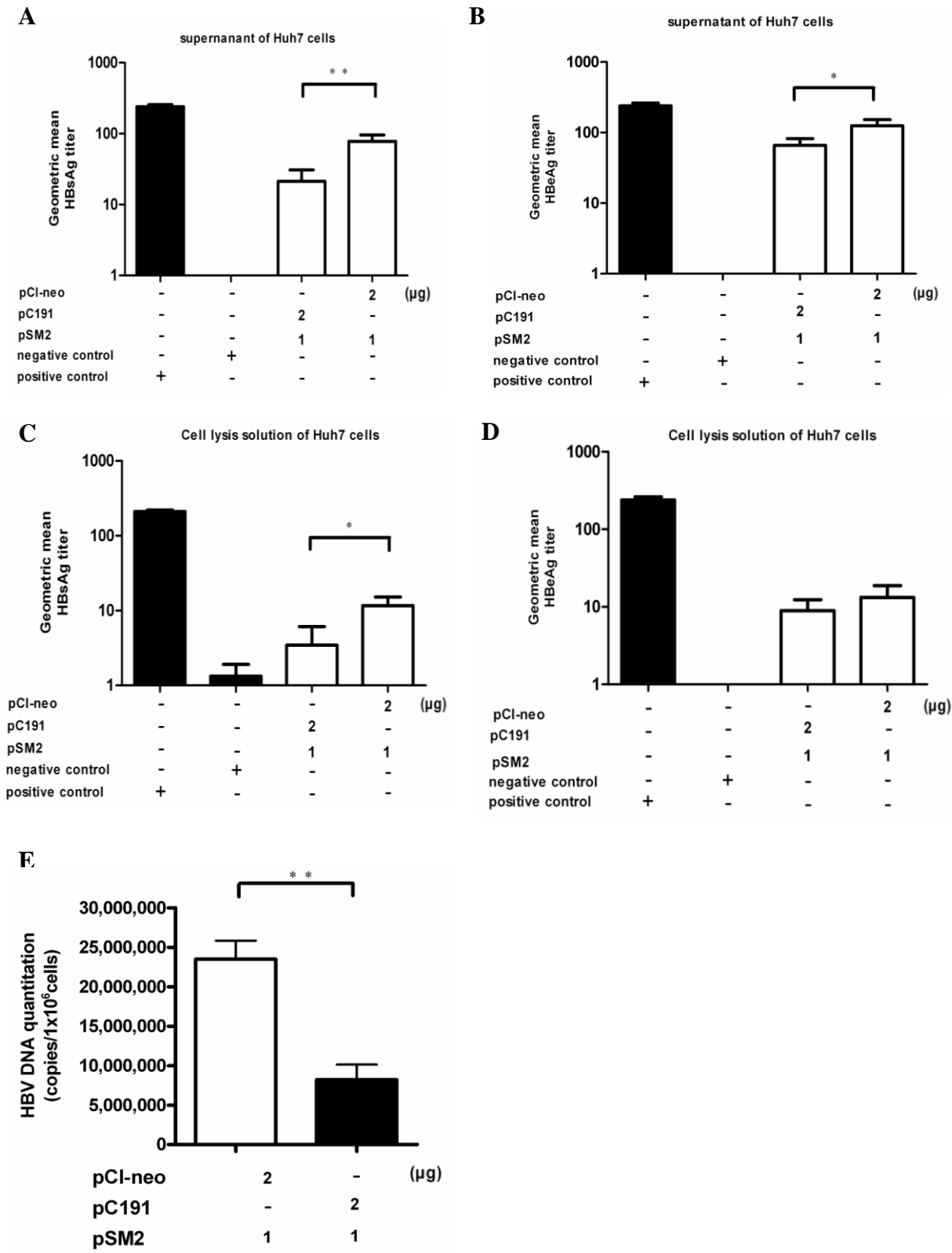

Supplement: Figure S1 — HBV replication and gene expression in Huh7 cells in the presence of HCV core protein. Huh7 cells were co-transfected with plasmids of pSM2 and pC191 or pCI-neo. The HBsAg and HBeAg titers in the culture supernatants (A, B) and cell lysats (C, D) were determined. The amounts of encasidated HBV DNA in transfected cells were determined by real time PCR (E). (PDF) [file pone.0045146.s001.pdf]

Supplemental Figure 2

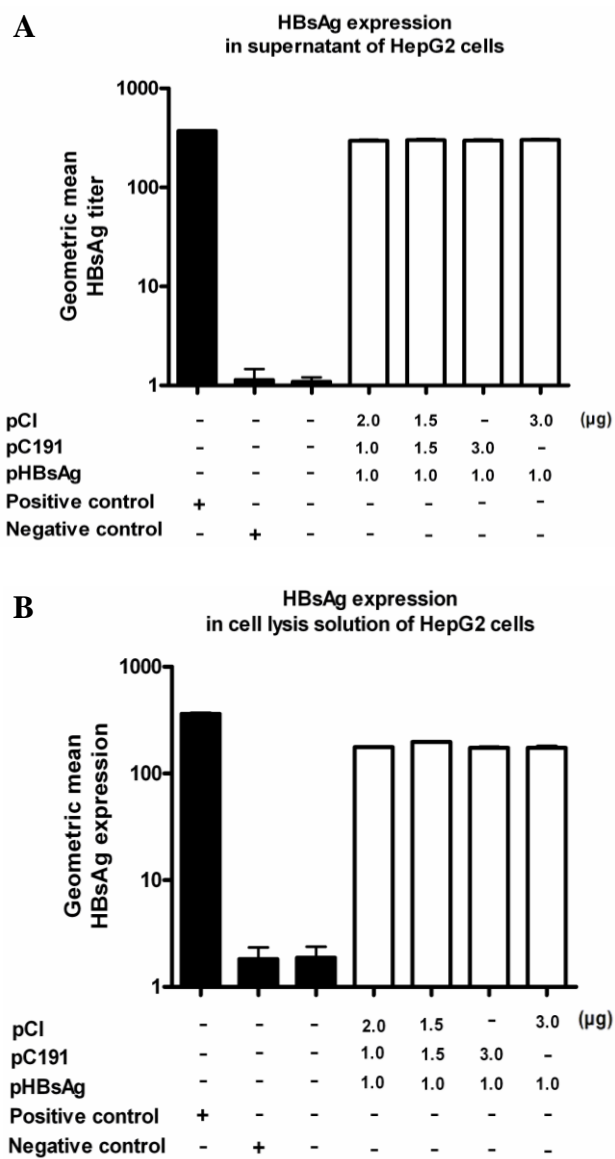

Supplement: Figure S2 — HBsAg expression in hepatoma cells in the presence of HCV core protein. HepG2 cells were co-transfected with plasmids pCI-neo, pHBsAg, or pC191 as indicated. HBsAg in the culture supernatants (A) and cell lysats (B) of transfected HepG2 cells were determined. (PDF) [file pone.0045146.s002.pdf]

Supplemental Figure 3

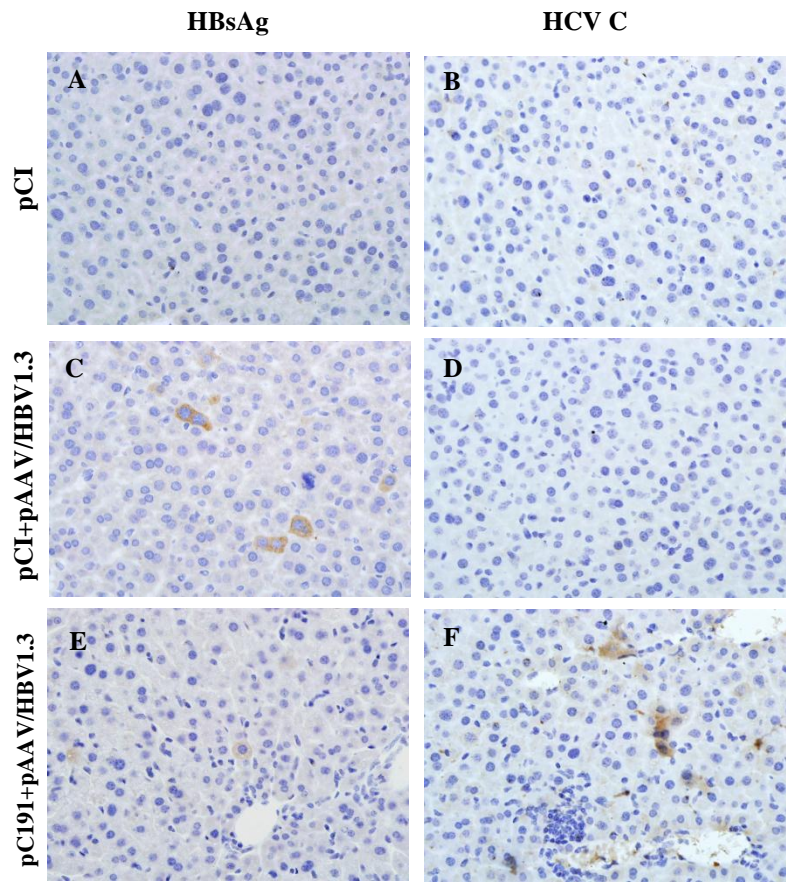

Supplement: Figure S3 — Immunohistochemistry staining analysis of HBsAg and HCV in mice liver. BALB/c (H-2d) mice were subjected to HI with different plasmids combinations. Liver were collected and subjected to immunohistochemistry staining with anti-HBsAg (Thermo) (A, C and D) and serum of HCV patient (B, D and F) at 72 h (magnification: 200 X ). (PDF) [file pone.0045146.s003.pdf]
